# Abnormal Cerebrovascular Reactivity in Patients with Parkinson's Disease

**DOI:** 10.1155/2015/523041

**Published:** 2015-06-16

**Authors:** Carlos Henrique Ferreira Camargo, Eduardo Antunes Martins, Marcos Christiano Lange, Henrique Alvaro Hoffmann, Jissa Jeanete Luciano, Marcelo Rezende Young Blood, Marcelo Derbli Schafranski, Marcelo Machado Ferro, Edmar Miyoshi

**Affiliations:** ^1^State University of Ponta Grossa, Avenida General Carlos Cavalcanti 4748, 84030-900 Ponta Grossa, PR, Brazil; ^2^Hospital de Clínicas, Federal University of Paraná, Rua General Carneiro 181, 80060-900 Curitiba, PR, Brazil

## Abstract

*Background*. Orthostatic hypotension (OH) is an important nonmotor manifestation of Parkinson's disease (PD). Changes in cerebrovascular reactivity may contribute to this manifestation and can be monitored using transcranial Doppler. *Objective*. To identify possible changes in cerebrovascular reactivity in patients with OH. *Methods*. Twenty-two individuals were selected and divided into three groups: with and without OH and controls. Transcranial Doppler was used to assess basal mean blood flow velocity, postapnea mean blood flow velocity, percentage increase in mean blood flow velocity, and cerebrovascular reactivity as measured by the breath-holding index. *Results*. PD patients had lower values of basal velocity (*p* = 0.019), postapnea velocity (*p* = 0.0015), percentage increase in velocity (*p* = 0.039), and breath-holding index (*p* = 0.04) than the controls. Patients with OH had higher values of basal velocity (*p* = 0.09) and postapnea velocity (*p* = 0.19) but lower values of percentage increase in velocity (*p* = 0.22) and breath-holding index (*p* = 0.32) than patients without OH. *Conclusions*. PD patients present with abnormalities in a compensatory mechanism that regulates cerebral blood flow. OH could be an indicator of these abnormalities.

## 1. Introduction

Parkinson's disease (PD) is characterized by slow degeneration of specific neurons in the enteric, peripheral, and central nervous system [1]. Analysis of lesions in PD by Braak et al. [2] showed that the disease progresses in six stages in a caudorostral direction, starting in caudal regions of the brain stem, such as the dorsal motor nuclei of the glossopharyngeal and vagus nerves and the anterior olfactory nucleus, and spreading to practically the whole cortex [3]. Based on these pathological findings and the clinical presentation of the disease, the definition of PD as only a motor disease is believed to be clearly inadequate. Dysautonomias are one of the most important nonmotor complications of PD [4], and orthostatic hypotension (OH) is quite a common complaint [5], occurring in around 40% of PD patients [6].

A drop in systemic arterial blood pressure is normally compensated for by a sympathetically mediated increase in vascular tone and cerebral vasodilation. PD patients, however, present with worse hemodynamic parameters because of degeneration of central and peripheral nuclei (brainstem, cerebral cortex, spinal cord, and autonomic ganglia [2]); baroreflex failure, with a reduction in the number of catecholaminergic neurons in the nucleus of the solitary tract [7]; diffuse cardiac noradrenergic denervation of the left ventricle [8]; abnormal pressure natriuresis and diuresis due to loss of specific neurotransmitters [9]; suboptimal release of norepinephrine when the patient stands up [10], with an increase in the number of *α*-adrenoreceptors in an attempt to control sympathetic dysfunction in this position [11]; and the presence of Lewy bodies in axons in the paravertebral sympathetic chain and the stellate ganglion [10]. Therefore, a decrease in sympathetic tone in PD patients with OH is well known and the mechanisms involved are well established, which is not yet the case for the mechanisms responsible for maintaining cerebral blood flow [12].

Transcranial Doppler (TCD) allows cerebral blood flow velocity (cBFV) and the contractility of cerebral vessels to be measured dynamically and with high temporal resolution [13]. An increase in the concentration of CO_2_ in the blood stream leads to vasodilation of the intracranial microcirculation, which can be observed in TCD as an increase in cBFV. This change in cBFV in response to a vasodilatory stimulus is known as cerebrovascular reactivity (CVR) [14]. Various techniques can be used to estimate CVR [15, 16], such as measurement of the percentage change in the mean blood flow velocity (mBFV) in the middle cerebral artery (MCA) between hyperventilation and inspiration of increasing concentrations of CO_2_ or the inspiration of 5% CO_2_. However, the technique based on the use of breath-holding as the vasodilatory stimulus is the most suitable as it is both practical and easy-to-use [16]. The breath-holding index (BHI) was first described by Ratnatunga and Adiseshiah [17], who observed that the change in the mBFV of the MCA after a period of apnea without prior forced inspiration divided by the apnea duration gave an estimate of the change in cerebral blood flow and therefore CVR. Markus and Harrison [16] showed that this methodology was equivalent to those based on inspiration of CO_2_ and also defined an ideal time and minimum apnea duration (30 and 15 seconds, resp.).

Despite the existence of these various approaches, to the authors' knowledge there are no studies that provide absolutely conclusive findings about the changes in CVR in PD patients. While the first studies to correlate the findings of TCD with OH and PD did not find any changes, more recent studies using other approaches found significant changes in CVR in patients with PD compared with controls. The present study is the first to use the BHI to show that these changes occur and, furthermore, is one of the few ones to compare OH patients with patients without OH rather than only with controls.

The aim of the present study was to identify possible changes in CVR measured using the BHI in patients with OH associated with PD.

## 2. Materials and Methods

The study sample consisted of 20 patients with a confirmed diagnosis of PD according to the UK Parkinson's Disease Society Brain Bank clinical diagnostic criteria [18] who were being followed up regularly at the Neurology Service at the Campos Gerais Regional University Hospital (HURCG) [19].

Patients with Parkinsonism-plus disease, Parkinsonism as an associated feature of heredodegenerative diseases, and secondary Parkinsonism were excluded, as were patients who were using dopamine agonists and those who refused to sign the voluntary informed-consent form.

The study was approved by the State University of Ponta Grossa (UEPG) Research Ethics Committee (COEP) (reference number FA 22591).

### 2.1. Clinical Assessment

Patients were evaluated clinically, neurologically, and for the presence of OH [19], which was defined as a drop of at least 20 mmHg in systolic blood pressure and/or a drop of at least 10 mmHg in diastolic blood pressure as a result of a change from a supine to a standing position after one minute [11]. Ultrasound studies of extracranial and intracranial blood flow were performed to exclude occlusive diseases.

Patients were then divided into two groups: those with OH (*n* = 9) and those without (*n* = 11). Two patients from the group with OH and five from the group without OH were not examined by TCD as they did not consent to proceed with the study. One patient from the group with OH and one from the group without OH were excluded from the study because of inadequate temporal acoustic windows. A control group (*n* = 11) was formed from healthy individuals recruited among the patients' relatives and hospital staff. The final study population therefore consisted of six patients in the group with OH, five in the group without OH, and eleven controls.

### 2.2. Transcranial Doppler (TCD)

TCD was carried out in a quiet, dimly lit room. Tests were carried out between 9 am and noon Brasília time. All the patients were required to lie down in the examination room for 5 minutes before the start of the test, which lasted between 15 and 20 minutes. Patients were instructed not to use the first dose of levodopa (L-dopa) in the morning. All tests were performed by a single researcher who had previous experience in TCD. The researcher was unaware of the clinical condition of each participant.

An S4-2 2 MHz sector-phased array transducer coupled to a Philips HD11 XE Ultrasound System (Philips©, Philips Medical Systems B.V., Netherlands) was used to assess the M1 segment (45–55 mm deep) of the MCA in both sides.

The tests started on the left MCA and the following information was collected: basal mean blood flow velocity (bBFV) in cm/s; postapnea mean blood flow velocity (aBFV) in cm/s; and duration of apnea in s. Throughout the procedure the transducer was kept in the place that had been initially identified as suitable for measuring the desired parameters. CVR (absolute value) was estimated using the BHI [16], which is given by the following formula:

(1) BHI=%IBFV/bBFVT,where  %IBFV=aBFV−bBFV·100,

where BHI = breath-holding index; aBFV = mean postapnea blood flow velocity; bBFV = basal mean blood flow velocity; *T* = duration of apnea in seconds. Values of BHI of less than 0.70 were considered abnormal [16]. The percentage increase in blood flow velocity (%IBFV) was analyzed.

### 2.3. Statistical Analysis

The statistical differences between the means of the groups were measured using the two-tailed Student's *t*-test for normal distributions and the Mann-Whitney test for nonnormal distributions. Fisher's exact test was used for categorical variables. The effect size was analyzed using the odds ratio for categorical variables and Cohen's *d* coefficient for continuous variables (0.2: weak effect; 0.5: moderate effect; and 0.8: strong effect). The results are given as mean ± standard deviation (SD) or as an odds ratio (OR) and 95% confidence interval (CI) (OR (95 %CI)). *p* values of less than 0.05 were considered statistically significant. The analysis was performed with MedCalc 5.1 (Belgium) (MedCalc version 11.5.1-MedCalc, Mariakerke, Belgium) and Microsoft Excel.

## 3. Results

### 3.1. Demographics

Of the patients with PD, six (54.54%) had OH, but only two (33.3%) of these patients presented with complaints compatible with OH. There were no significant differences in age (67.36 ± 11.73 versus 64.727 ± 11.867, *p* = 0.61) or gender between the control group and patients with PD. The ratio of men to women was 2.67 : 1 (eight men, 72.74%, and three women, 27.27%). The control group consisted of seven men (63.64%) and four women (36.36%), giving a ratio of men to women of 1.75 : 1 (Table 1). There were no significant differences in terms of L-dopa use, age, and gender between patients with and without OH (Table 2).

### 3.2. CVR

Of all the patients with PD, six (54.5%) had an abnormal BHI. Four of these (66.7%) were from the group with OH and two (40%) from the group without OH, *p* = 0.57 (3 (0.25–35.33)). There was a greater percentage of abnormal BHI values in the PD group (54.5%) than in the control group (27.3%), *p* = 0.04 (4.75 (1.07–21.14)).

### 3.3. Relationship between TCD Findings and OH

Patients with PD had lower values of bBFV, aBFV, %IBFV, and BHI than controls (Table 3). Individuals with OH had lower values of bBFV, aBFV, %IBFV, and BHI than controls (Table 4). PD patients without OH also had values of bBFV and aBFV that were significantly lower than in controls (Table 5).

When the results for PD patients with and without OH were compared, the former had higher values of bBFV (49.93 ± 12.36 versus 43.39 ± 9.65, *p* = 0.09; Cohen's *d* = 0.59) and aBFV (61.27 ± 12.26 versus 56.25 ± 13.73, *p* = 0.19; Cohen's *d* = 0.39) and lower values of %IBFV (24.65 ± 14.08 versus 30.54 ± 20.64; *p* = 0.22; Cohen's *d* = 0.33) and BHI (0.88 ± 0.5 versus 1.04 ± 0.98; *p* = 0.32; Cohen's *d* = −0.21); however, these results were not statistically significant.

## 4. Discussion

The prevalence of OH in the study sample was 54.54%, and 33% of the patients were symptomatic. These figures agree with the prevalence reported in the literature of 40–60% among PD patients [6] with only 20% reporting some symptoms [20].

The present study has shown that PD patients have altered CVR compared with controls. To our knowledge, it is the first study to establish a correlation between BHI and PD. Niehaus et al. [21], using TCD and the tilt-table test, reported a small increase in heart rate (HR) and a greater, more prolonged drop in arterial blood pressure (ABP) in PD patients who were tilted close to the upright position. However, no changes in cBFV were observed in these patients, whose CA was similar to that of the control group. Angeli et al. [22] observed a hypotensive response to orthostatic stress, with intracranial vasodilation and lower diastolic pressure in PD patients monitored with TCD during tilt-table testing. Gurevich et al. [12] compared CA and CVR in PD patients with multiple system atrophy (MSA) and pure autonomic failure (PAF) using TCD, the acetazolamide test, and the tilt-table test but failed to find any change in CVR.

Our findings of altered CVR and lower cBFV agree with more recent studies that used TCD to analyze cerebral hemodynamics in PD patients [23, 24]. Vokatch et al. [11] used TCD and thigh cuffs to assess CA and found striking differences in mBFV between controls and PD patients, especially after a reduction in blood pressure, providing strong evidence of impaired CA in patients with this disorder. Furthermore, L-dopa did not appear to influence the changes in these parameters. Using the cold pressor test, Tsai et al. [24] found similar changes in cBFV. However, they did not take into account whether their patients were using L-dopa or not in their study. Bouhaddi et al. [25] used TCD and the tilt-table test to compare PD patients taking and not taking L-dopa and concluded that this medication could further impair autonomic control of heart rate and blood pressure.

Previously published studies of OH in PD patients [11, 21, 22, 24] that investigated CA did not reach definitive conclusions about its impact on cBFV or the possible impact of the use of L-dopa on autonomic dysfunction [25].

In the present study, the values of bBFV, %IBFV, and BHI were lower in PD patients with OH than in controls. CVR can be estimated by measuring the change in cBFV in response to vasodilatory stimuli [14]. bBFV and aBFV represent cBFV at baseline and after a normally vasodilatory stimulus, respectively, and %IBFV is the relative difference between them [16]. Previous studies did not identify these differences even though they used methods that were theoretically similar to the method using breath-holding as a vasodilatory stimulus [16]. This probably occurred because these studies used 8% CO_2_ instead of BHI.

The probable pathophysiological explanation for the TCD findings observed in the present study is that hemodynamically compromised tissue is supplied by arterioles that are already maximally, or near maximally, dilated. A stimulus that is normally vasodilatory is therefore unable to produce an adequate response [16]. It appears that OH patients have greater degeneration of the sympathetic nervous system, leading to significant hemodynamic impairment. Hence, all the cerebrovascular reserve capacity may be used up under basal conditions, and when an increase in blood supply is required these values cannot be compensated for, resulting in the changes observed in aBFV, %IBFV, and BHI, a drop in pressure and, consequently, OH. PD patients without OH probably have less severe autonomic impairment, which is reflected in a lower flow velocity under basal conditions [23, 25]. Therefore, because CVR is less affected in these patients, their response to changes in blood supply requirements is normal and does not lead to OH. A similar hypothesis has already been proposed by Haubrich et al. [26], although they concluded that autoregulatory mechanisms in PD patients were the same as in healthy individuals.

This study has a number of limitations. Firstly, we assumed that L-dopa does not influence CVR in PD patients [11, 23]. This could have been confirmed by carrying out two sets of tests, one with and the other without the medication. Secondly, as BHI and similar indexes [16] have only been tested on patients without any neurological condition to estimate CA, it would be useful to investigate these indexes in PD patients. Lastly, as our sample was small, a study with more patients and controls should be carried out to confirm the conclusions.

We have shown that PD patients have abnormal cBFV, indicating that cerebral hemodynamic alterations may also be present in these patients. Individuals with PD and OH appear to have altered CVR and great difficulty in satisfying tissue requirements under nonbasal conditions, which could explain the clinical findings for these individuals. Nevertheless, further studies are required to confirm these results.

## Figures and Tables

**Table 1:** Comparison of controls and PD patients.

	PD	Controls	*p*
Mean age (years)	67.36 ± 11.73	64.727 ± 11.867	*p* = 0.606

Gender			*p* = 1
M	8	7	
F	3	4	

Note: PD = Parkinson's disease; OH = orthostatic hypotension.

**Table 2:** Clinical and demographic data for the PD patients with and without orthostatic hypotension.

	PD without OH	PD with OH	*p*
Mean age (years)	62 ± 14.16	71.8 ± 7.89	*p* = 0.657

Gender			
M	3	5	
F	2	1	

Age of onset (years)	56 ± 16.58	68.5 ± 7.4	*p* = 0.176

Disease duration (years)	6 ± 3.54	3.33 ± 1.03	*p* = 0.169

Duration of L-dopa use (years)	4.3 ± 3.03	3.17 ± 1.33	*p* = 0.471

Duration of disease before L-dopa started (years)	1.7 ± 2.11	0.17 ± 0.41	*p* = 0.181

Predominance of symptoms			
Predominantly akinetic-rigid	4	4	*p* = 1
Predominant tremor	1	2	*p* = 1

Dizziness and falls			*p* = 0.242
Yes	2	5	
No	3	1	

UPDRS	11,6 ± 7,23	15,167 ± 13,41	*p* = 0.304

Hoehn and Yahr [1]	1,8 ± 1,1	2 ± 1,1	*p* = 0.385

Note: PD = Parkinson's disease; OH = orthostatic hypotension.

**Table 3:** Cerebral hemodynamic data for PD patients and controls.

	PD	Controls	*p*-Cohen
bFV (cm/s)	46.41 ± 11.38	54.42 ± 12.03	0.019
aBFV (cm/s)	58.88 ± 12.9	75.15 ± 20.07	0.0015
%IBFV (%)	27.45 ± 17.31	37.94 ± 20.53	0.039
BHI	0.96 ± 0.75	1.35 ± 0.74	0.04

Note: PD = Parkinson's disease; bBFV = basal mean blood flow velocity (cm/s); aBFV = mean postapnea blood flow velocity (cm/s); %IBFV = percentage increase in mean velocity during breath-holding (%); BHI = breath-holding index.

**Table 4:** Cerebral hemodynamic data for PD patients with OH and controls.

	PD and OH	Controls	*p*-Cohen
bBFV (cm/s)	49.93 ± 12.36	54.42 ± 12.03	0.16
abFV (cm/s)	61.27 ± 12.26	75.15 ± 20.07	0.02
%IBFV (%)	24.65 ± 14.08	37.94 ± 20.53	0.032
BHI	0.88 ± 0.5	1.35 ± 0.74	0.03

Note: PD = Parkinson's disease; OH = orthostatic hypotension; bBFV = basal mean blood flow velocity (cm/s); aBFV = mean postapnea blood flow velocity (cm/s); %IBFV = percentage increase in mean velocity during breath-holding (%); BHI = breath-holding index.

**Table 5:** Cerebral hemodynamic data for PD patients without OH and controls.

	PD without OH	Controls	*p*-Cohen
bBFV (cm/s)	43.39 ± 9.65	54.42 ± 12.03	0.008
abFV (cm/s)	56.25 ± 13.73	75.15 ± 20.07	0.057
%IBFV (%)	30.54 ± 20.64	37.94 ± 20.53	0.23
BHI	1.04 ± 0.98	1.35 ± 0.74	0.16

NB: PD = Parkinson's disease; OH = orthostatic hypotension; bBFV = basal mean blood flow velocity (cm/s); aBFV = mean post-apnea blood flow velocity (cm/s); %IBFV = percentage increase in mean velocity during breath-holding (%); BHI = breath-holding index.
